# Potential Role of ANGPTL4 in the Cross Talk between Metabolism and Cancer through PPAR Signaling Pathway

**DOI:** 10.1155/2017/8187235

**Published:** 2017-01-15

**Authors:** Laura La Paglia, Angela Listì, Stefano Caruso, Valeria Amodeo, Francesco Passiglia, Viviana Bazan, Daniele Fanale

**Affiliations:** ^1^ICAR-CNR, National Research Council of Italy, 90146 Palermo, Italy; ^2^Department of Surgical, Oncological and Oral Sciences, Section of Medical Oncology, University of Palermo, 90127 Palermo, Italy; ^3^Génomique Fonctionnelle des Tumeurs Solides, INSERM, UMR 1162, 75010 Paris, France; ^4^Samantha Dickson Brain Cancer Unit, UCL Cancer Institute, University College London, London WC1E 6DD, UK

## Abstract

The angiopoietin-like 4 (ANGPTL4) protein belongs to a superfamily of secreted proteins structurally related to factors modulating angiogenesis known as angiopoietins. At first, ANGPTL4 has been identified as an adipokine exclusively involved in lipid metabolism, because of its prevalent expression in liver and adipose tissue. This protein regulates lipid metabolism by inhibiting lipoprotein lipase (LPL) activity and stimulating lipolysis of white adipose tissue (WAT), resulting in increased levels of plasma triglycerides (TG) and fatty acids. Subsequently, ANGPTL4 has been shown to be involved in several nonmetabolic and metabolic conditions, both physiological and pathological, including angiogenesis and vascular permeability, cell differentiation, tumorigenesis, glucose homoeostasis, lipid metabolism, energy homeostasis, wound healing, inflammation, and redox regulation. The transcriptional regulation of ANGPTL4 can be modulated by several transcription factors, including PPAR*α*, PPAR*β*/*δ*, PPAR*γ*, and HIF-1*α*, and nutritional and hormonal conditions. Several studies showed that high levels of ANGPTL4 are associated with poor prognosis in patients with various solid tumors, suggesting an important role in cancer onset and progression, metastasis, and anoikis resistance. Here, we have discussed the potential role of ANGPTL4 in mediating the cross talk between metabolic syndromes, such as diabetes and obesity, and cancer through regulation of its expression by PPARs.

## 1. Peroxisome Proliferator-Activated Receptors (PPARs): Structure and Functions

Peroxisome proliferator-activated receptors (PPARs) are ligand-activated transcription factors belonging to the steroid hormone receptor superfamily, identified for the first time in 1990 by Issemann and Green [1]. There are three distinct PPAR subtypes, PPAR*α*, PPAR*β*/*δ* (also known as PPAR*β* or PPAR*δ*), and PPAR*γ*, encoded by specific genes located on different chromosomes. Although these three members show a significant homology, they differ from each other for tissue distribution, affinity for ligands, and biological functions [2]. All subtypes are activated by endogenous ligands and participate in the regulation of several genes involved in glucose and lipid metabolism. However, other specific PPAR actions are limited to specific tissue types [3, 4]. PPAR*α*, the first PPAR to be cloned, is highly expressed in tissues characterized by elevated fatty acid oxidation such as liver, heart, skeletal muscle, brown adipose tissue, kidney, adrenal gland, and intestinal mucosa, where it plays a key role in the fatty acid catabolism [5, 6]. PPAR*β*/*δ* is expressed in most of human tissues, mainly in the liver, adipose tissue, skeletal muscle, heart, brain, kidney, skin, and intestine, characterized by an increased lipid metabolism. However, the function of this isoform remains to be elucidated [7–10]. PPAR*γ* is highly expressed in white and brown adipose tissue (WAT and BAT) and plays a pivotal role in the regulation of adipogenesis, fat storage, and glucose metabolism [11–15]. In addition, PPAR*γ* also regulates the expression of proinflammatory cytokines, such as tumor necrosis factor-*α* (TNF-*α*), as well as genes involved in insulin sensitivity. For this reason, PPAR*γ* is the main target of thiazolidinediones (TZDs), a class of drugs used to improve lipid and glucose metabolism in type 2 diabetes [16, 17].

**Figure 1:**
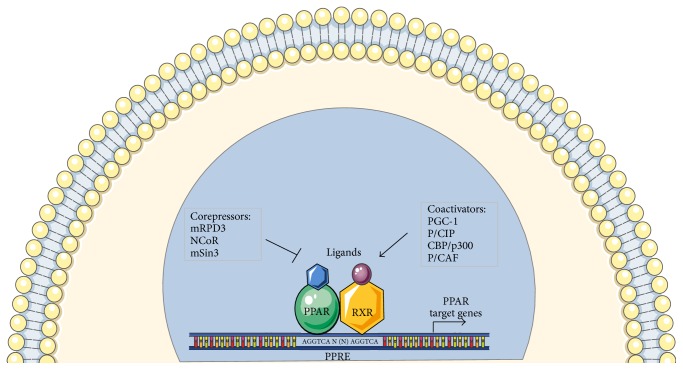
Interaction between PPARs and PPRE. The figure shows PPAR structure and related coactivator and corepressor molecules involved in activation and repression mechanisms. The activation signaling of PPAR-RXR heterodimer and PPRE allows the expression modulation of target molecules such as ANGPTL4.

PPARs show a DNA binding domain (DBD) in the N-terminal and a ligand binding domain (LBD) in the C-terminal separated by a hinge region acting as a docking site for cofactors [18]. Three PPAR isoforms exhibit an 80% homology and are more divergent in the LBD, confirming their different response to various ligands. After activation by endogenous or synthetic ligands, PPARs undergo a conformational change that causes the translocation to the nucleus and the heterodimerization with another nuclear receptor, the retinoid X receptor (RXR) [19]. The PPAR-RXR heterodimer then binds a DNA portion in the promoter region of target genes, called peroxisome proliferator response element (PPRE), modulating the expression of several genes involved in different physiological or pathological processes [20]. Interestingly, the PPAR functions also depend on the binding with different coactivator and corepressor proteins [21]. Indeed, after interaction with agonists, the conformational change of the PPAR structure causes also the attachment of coactivators and detachment of corepressors. Usually, PPAR-RXR heterodimers are packed with a corepressor molecule in PPRE and the binding with ligands causes an exchange of corepressors for coactivators. One of the more studied PPAR corepressors is histone deacetylase (HDAC). Among the different coactivators, there are PGC-1, p300, and CREB that are involved in regulation of metabolism as well as in cancer development [22, 23] (Figure 1).

## 2. Angiopoietin-Like 4 (ANGPTL4): Structure and Expression Patterns

The angiopoietin-like 4 (ANGPTL4) protein was discovered for the first time in 2000 by three independent research groups. They simultaneously identified this molecule as a fasting-induced adipose factor (Fiaf) in different tissues. ANGPTL4 is mainly expressed in liver and adipose tissue, as shown by Kersten et al. [24] that highlighted its upregulation in these tissues during fasting and a PPAR-dependent mRNA regulation, using PPAR*α*/*γ* wild-type and mutant mice. Also, Kim et al. [25] identified a novel angiopoietin-like protein mainly expressed in hepatocytes. Finally, Cliff Yoon et al. [26] proved the regulative relation between PPAR proteins and ANGPTL4, demonstrating that ANGPTL4 is a target of PPAR*γ* in adipose tissue.

**Figure 2:**
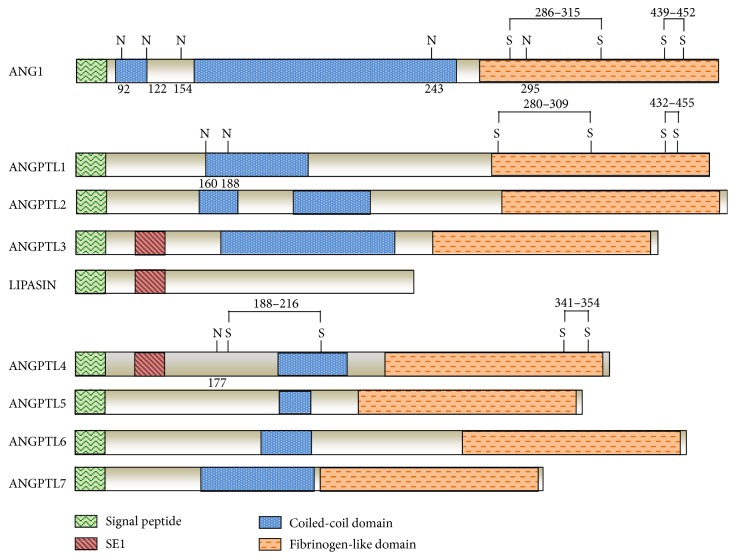
Structural organization and homology between ANGPTL family members. Signal peptide domain is shown in green, specific epitope 1 (SE1) (region present in ANGPTL3 and ANGPTL4 important for binding LPL and inhibiting its activity in vitro and in vivo) in purple red, coiled-coil domain in blue, and N-terminal fibrinogen-like domains in orange. Glycosylation sites (N) are shown at positions 92, 122, 154, 243, and 295 for ANG1; positions 160 and 188 for ANGPTL1; and position 177 for ANGPTL4. Disulfide bonds (SS) are shown at positions 286–315 and 439–452 for ANG1; 280–309 and 432–455 for ANGPTL1; 188–216 and 341–354 for ANGPTL4.

ANGPTL4 belongs to a superfamily of secreted proteins structurally related to factors modulating angiogenesis known as angiopoietins (ANG). This protein family includes eight members encoded by eight genes (ANGPTL1-8) identified in humans and mice, except ANGPTL5, that is a human orthologue [25, 27–29]. Only recently, in 2012, a new feeding-induced hepatokine was identified and called RIFL/lipasin/ANGPTL8 (also known as betatrophin) [30–33]. Just in 2015, the HUGO gene nomenclature defined the official name of the protein as ANGPTL8 [34]. Like angiopoietins, all angiopoietin-like proteins (ANGPTLs) exhibit a C-terminal fibrinogen-like globular domain and an N-terminal coiled-coil domain [35] except for ANGPTL8. Indeed, this last ANGPTL family member is considered an atypical member, since it lacks the main structural features present in all other proteins of the group, such as the fibrinogen-like domain, glycosylation sites, and amino acids requested for formation of disulfide bonds [32] (Figure 2). Unlike the angiopoietins, ANGPTLs are considered orphan ligands, as they do not bind to either the angiopoietin receptor tyrosine kinase Tie2 or the related protein Tie1 [36–38].

The first four family members (ANGPTL1-4) and ANGPTL6/angiopoietin-related growth factor (AGF) have been shown to modulate angiogenesis. ANGPTLs 3, 4, 5, and 8 and ANGPTL6/AGF seem to be involved also in regulation of other processes such as lipid metabolism and glucose and energy homeostasis [39–46]. Another study showed that ANGPTLs 3 and 4 control lipid metabolism by inhibiting the activity of lipoprotein lipase (LPL) [47], an enzyme responsible for hydrolysis of triglycerides (TG) contained in lipoproteins, such as chylomicrons and very low-density lipoproteins (VLDL), fatty acids, and cholesterol, whereas ANGPTL6/AGF antagonizes obesity and related metabolic diseases, including insulin resistance, by enhancing systemic energy expenditure [45].

ANGPTLs show different tissue expression patterns. ANGPTL1 is mostly detected in liver, heart, skeletal muscle, kidney, and vessel-rich endocrine organs (adrenal glands, thyroid, and pituitary gland) but also to a lesser extent in uterus and gastrointestinal tract [48]. ANGPTL2 shows high expression levels in heart, stomach, adipose tissue, skeletal muscle, and uterus [49], whereas ANGPTL3 is predominantly expressed in liver [50, 51]. ANGPTL4 shows ~30% of sequence homology with ANGPTL3. It is abundantly present in the liver, adipose tissue, and skeletal muscle and, to a lesser extent, in placenta, small intestine, heart, and pituitary gland [52–56]. ANGPTL5 is mainly expressed in adult human heart [57], whereas ANGPTL6/AGF expression is restricted to liver and plasma [58]. Lastly, ANGPTL7 exhibits high expression levels in the cornea, neural tissues, and trabecular meshwork as well as uterine endometrial cancer and melanoma [59].

The human gene encoding ANGPTL4 is evolutionarily conserved among species and shares a sequence homology of ~77% with mouse. It is located on chromosome 19p13.3 and consists of seven exons encoding a 406-amino acid glycoprotein. Like other proteins of the ANGPTL family, ANGPTL4 contains a C-terminal fibrinogen-like domain (cANGPTL4) and an N-terminal coiled-coil folding domain (nANGPTL4), in which a highly hydrophobic region that acts as a signal peptide for protein secretion is present. In addition, it exhibits several potential N- and O-glycosylation sites and was found to be N-glycosylated at amino acid position 177 [37, 60]. The same domains of full-length ANGPTL4 (flANGPTL4) protein structure were found in plasma [61, 62]. Higher-order oligomeric structures can be formed by native flANGPTL4 through the formation of intermolecular disulfide bonds. ANGPTL4 contains several conserved cysteine residues that contribute to the formation of variable-sized multimeric structures. The N-terminal oligomerization of ANGPTL4 requires the presence of two cysteine residues (Cys-76 and Cys-80) in the N-terminal portion [63].

Different studies showed that nANGPTL4 domain is used to modulate lipid metabolism, whereas cANGPTL4 domain may be a modulator of tumorigenesis process [54]. Indeed, ANGPTL4 can exert its function of LPL activity inhibitor thanks to an oligomerization process mediated by the N-terminal region responsible for its assembly into tetrameric or dimeric structures [64–66]. It was hypothesized that LPL blockage is due to 12 highly conserved amino acids that are near the N-terminus of the protein. Indeed, mutations in three polar amino acid residues within this region abolished the ability of ANGPTL4 to inhibit LPL [66]. Experimental evidence showed that several proprotein convertases, including furin, PC5/6 (proprotein convertase 5/6), and PCSK3 (proprotein convertase subtilisin/kexin type 3), catalyze the proteolytic processing of the human flANGPTL4 protein via recognition of a specific amino acid sequence, causing the release of the N-terminal region and monomeric C-terminal portion [66, 67]. ANGPTL4 is cleaved in a tissue-dependent manner and can be secreted into the bloodstream from adipose tissue and liver in native and cleaved, glycosylated, and oligomerized isoforms. In humans, the truncated form is secreted from liver, whereas the full-length form is released from adipose tissue [61, 62]. Furthermore, ANGPTL4 has been shown to bind to heparin sulfate proteoglycans and interact with ECM (extracellular matrix) proteins, by inhibiting endothelial cell adhesion and migration and altering actin cytoskeleton [68–70].

The transcriptional regulation of ANGPTL4 and its resulting expression can be determined by several transcription factors, including PPAR*α*, PPAR*γ*, and HIF-1*α*. A deeper investigation about transcription activation of ANGPTL4 and all these TFs was done by Inoue et al. [71]. Starting from the evidence that all these factors are important angiogenic molecules, the authors assessed whether there could be a synergic action mechanism of these two different signals in stimulating ANGPTL4 as angiogenesis-related target gene. Indeed, microarray and ChiP-seq analyses showed a cross-enhancement of ANGPTL4 expression dependent on the conformational proximity of two response elements [71].

Different evidences showed that upregulation of ANGPTL4 expression is strongly linked to fasting in a variety of tissues [72, 73]. Different actors likely mediate the “fasting effect” [74]. Several findings showed that PPARs nuclear receptors induce an increase in ANGPTL4 expression. Glucocorticoids, whose circulating levels are high during fasting, also seem to mediate this event [75–78], and, in addition to fasting, chronic caloric restriction or free fatty acids (also called NEFA) have been shown to increase plasma ANGPTL4 levels [56]. Finally, some studies conducted on human myofibroblasts, using genome-wide transcriptional profiling technology, revealed that human ANGPTL4 expression might be synergistically induced by the functional interactions of TGF-*β* and PPAR *β*/*δ* signaling [79].

Since ANGPTL4 expression was found mainly in liver and adipose tissue, this molecule was classified, at first, as an adipokine exclusively involved in lipid metabolism. Afterwards, thanks to a large number of studies, this protein has been shown to have a highly multifaceted role, since it is involved in several nonmetabolic and metabolic conditions, both physiological and pathological, including angiogenesis and vascular permeability, cell differentiation, tumorigenesis, glucose homoeostasis, lipid metabolism, energy homeostasis, wound healing, inflammation, and redox regulation [80].

## 3. ANGPTL4: A Regulative Role in Glucose and Lipid Metabolism

As previously introduced, among the “pleiotropic” roles of ANGPTL4, greater attention was focused on its involvement in glucose and lipid metabolism regulation [81]. A positive correlation between increased ANGPTL4 and NEFAs levels in plasma of healthy subjects after dietary regimens has been shown. Conversely, the negative energy balance caused by fasting increases the hydrolysis of intracellular TG in adipocytes and other peripheral tissues. This leads to an increase in plasma NEFA levels [82].

Another recent work by Robciuc et al. showed the involvement of ANGPTL4 in mediating the PPAR*δ* effects on LPL activity in fatty acid (FA) uptake, whereas the effect of PPAR*δ* activation on *β*-oxidation is independent of ANGPTL4 [83]. Interestingly, the authors investigated also the role played by ANGPTL4 in regulating LPL activity, not only at the level of the surface of capillaries, highlighting the intracellular lipase degradation [83]. Other studies tried to better assess the cellular localization and molecular mechanisms underlying ANGPTL4 role in lipid metabolism, as reported by Dijk et al. [84]. These authors performed ex vivo and in vivo studies on adipocytes and adipose tissue from* wild-type* and ANGPTL4^−/−^mice, showing that ANGPTL4 stimulates LPL processing in the endoplasmic reticulum (ER) leading to its intracellular degradation [84]. Another study proposed that LPL regulation by ANGPTL4 occurs at cell surface [85].

The homeostasis of lipid metabolism is promoted through the intervention of lipases, enzymes that counterbalance LPL activity. Indeed, they hydrolyze stored TG, allowing adipocytes to release FA. Starting from these evidences, an interesting work by Koliwad et al. [78] showed that ANGPTL4 is a direct glucocorticoid receptor (GR) target and is involved in GR-dependent TG metabolism. Indeed,* ANGPTL4-null *mice showed lower plasma TG levels and increased ability to gain weight compared to mice overexpressing the gene, suggesting a role of ANGPTL4 in modulating TG homeostasis by regulation of its expression [78].

Lichtenstein et al. [73] have well explained the correlation between ANGPTL4 role and inhibition of LPL-mediated plasma TG lipolysis. Thanks to studies derived from transgenic mice, the molecular mechanisms underlying mouse blood TG were deeply revealed. Normally, LPL monomer is associated with the N-terminal domain of ANGPTL4 protein, thus shifting the balance between LPL dimers and monomers towards the latter, causing LPL inhibition and, consequently, determining the alteration of TG clearance from the plasma and FFA uptake decrease into the peripheral tissues [73].

**Figure 3:**
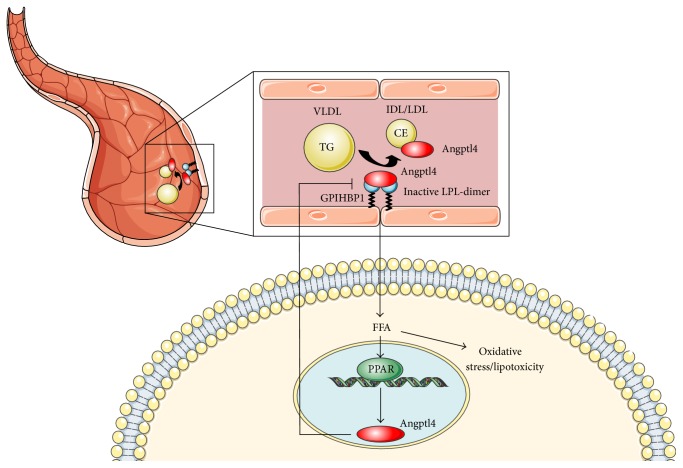
Molecular mechanism governing ANGPTL4-mediated TG hydrolysis. LPL monomer is linked to nANGPTL4 protein fraction, thus shifting the balance between LPL dimers and monomers towards the latter. As a consequence, LPL is inhibited and this causes the alteration of TG clearance from the plasma and uptake decrease of FFA into the peripheral tissues. The intervention of ANGPTL4 on LPL causes also the reduction of LPL affinity for GPIHBP1 protein.

Another mechanism by which ANGPTL4 inhibits LPL was proposed by Chi et al. These authors showed that ANGPTL4 can bind and inactivate LPL complexed to GPIHBP1. Therefore, the ANGPTL4-mediated LPL inactivation greatly reduces the affinity of LPL for GPIHBP1 [86] (Figure 3).

Considering the discussed role about ANGPTL4 in lipid metabolism, a possible link of this angiopoietin-like protein with obesity was investigated. Different murine models were proposed, highlighting a significant role of this protein in central regulation of energy metabolism [87]. More recently, Robciuc et al. carried out an interesting study on homozygous twins, in which a positive correlation between ANGPTL4 expression levels, adipose tissue hormone-sensitive lipase (LIPE), and CGI-58 gene was shown, supporting the hypothesis of the ANGPTLs' involvement in promoting lipolysis of adipocytes [88].

As previously reported, ANGPTL4 is mainly expressed in tissues such as WAT, liver, and skeletal muscle. A recent work by Alex et al. [89] highlighted protein expression also in human colon adenocarcinoma cells. The authors investigated the role of short-chain fatty acids (SCFA) in HT29 and T48 cell lines and showed an induction of ANGPTL4 synthesis by SCFA through PPAR*γ* receptor activation [89]. Furthermore, long-chain fatty acids have been shown to induce ANGPTL4 production and secretion by PPAR*δ* activation in skeletal muscle cells [90].

**Figure 4:**
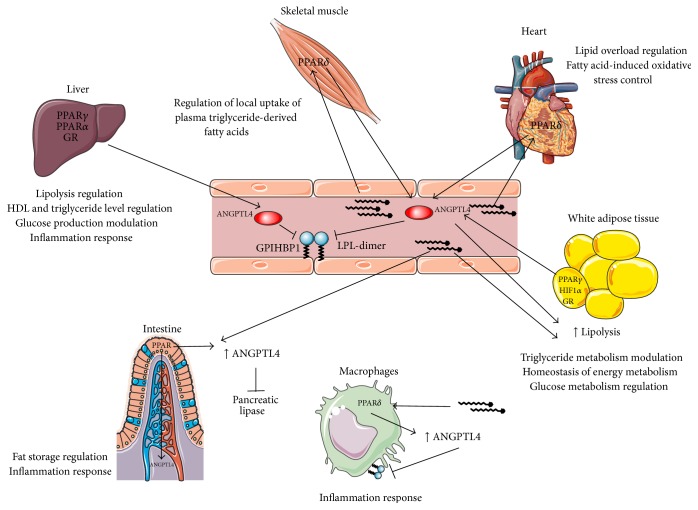
Regulation of expression and role of ANGPTL4 in lipid metabolism. In WAT, fasting induces ANGPTL4 expression through the action of different molecules such as PPARs, HIF-1*α*, and GR. The protein stimulates TG degradation via LPL inhibition. In liver, PPAR isoforms and GR stimulate ANGPTL4 expression. In this district, ANGPTL4 acts in part on hepatic LP and, in part, is released into the bloodstream, acting on LPL of peripheral tissues. In skeletal muscle, heart, and macrophages, FAs induce ANGPTL4 by PPAR*δ* activation. Also in intestine, FAs stimulate ANGPTL4 expression via one of the PPARs. ANGPTL4 produced by enterocytes is thus released towards the lumen and inhibits pancreatic LP.

As many scientific papers showed, several proteins, including ANGPTL4, inhibit LPL. A recent study focused the attention on the regulation mechanisms of another lipase, that is, the pancreatic lipase (PL). Interestingly, Mattijssen et al. suggested the involvement of ANGPTL4 also in endogenous inhibition of dietary lipids, through knockout mice experiments [91] (Figure 4).

However, ANGPTL4 is not the only angiopoietin-like family member involved in maintaining energy metabolism homeostasis. Other two members, such as ANGPTL3 and ANGPTL8, can inhibit LPL activity by affecting plasma TG levels. Indeed, different works showed overexpression of these proteins in mice and humans with increased plasma TG levels. Conversely, mutant mice carrying a loss-of-function of ANGPTLs exhibited low plasma TG levels [32, 92–94], although all three proteins show different tissue expression patterns and are regulated by different stimuli. This has led to the hypothesis that they are active during different metabolic states.

A recent work by Zhang proposed an ANGPTL3-4-8 molecular model to explain TG trafficking specifically in cardiac and skeletal muscles [95]. The model suggests that feeding can induce ANGPTL8, resulting in the activation of the ANGPTL3-8 pathway. This causes LPL inhibition and increase in plasma levels of TG which can be stored in WAT. In this district, decreases in ANGPTL4 concentration allow an increased LPL activity. The opposite scenario is presented during fasting. The impact of the ANGPTLs on plasma lipid levels has led to considering them as therapeutic targets for dyslipidemia [96].

Decreased plasma levels of this angiopoietin-like protein have been detected also after insulin induction. A recent study focused on the evaluation of the systemic effect of insulin on LPL and its regulative machinery in subjects with a different tolerance degree to insulin and showed a decrease in the adipose tissue ANGPTL4 expression in type 2 diabetes mellitus patients and healthy subjects [97].

Other evidences concerning the ANGPTL4 role in glucose metabolism regulation were reported in different studies on transgenic mice, where the decrease of blood glucose, improvement of glucose tolerance, and induction of hyperlipidemia and hepatic steatosis have been linked to the protein [44, 98]. More recently, other studies carried out on humans and animal models suggested the involvement of ANGPTL4 in nephrotic syndrome, revealing that ANGPTL4 acts by linking proteinuria and hypertriglyceridemia through negative feedback loops [99].

## 4. Role of ANGPTL4 in Cancer

The analysis of the different components of the tumor microenvironment and their cross talk have been the focus of the research of many molecular laboratories, especially since a lot of studies showed the key role played by tumor microenvironment in cancer development and progression. In vivo and in vitro models affirmed the ability of different molecular factors belonging to the tumor microenvironment in regulating cell–cell and cell–matrix communications, cell migration, invasion, and metastatic dissemination, but also in conditioning drastically the efficacy of antitumor therapies [100–102].

Currently, we know well that the malignance grade of a tumor is related to several factors, including genomic instability, heterogeneity (cells types such as fibroblasts, endothelial cells, pericytes, and immune cells), and composition of the microenvironment, which has been shown to change in different cancer types and also among different patients harbouring the same tumor histotype.

To date, the role of ANGPTL4 in cancer progression is not well defined, and there is still some controversy in the literature indicating the need of more studies addressing this interesting topic.

Several studies identified the presence of ANGPTL4 in various solid tumors, such as breast cancer, colorectal cancer, prostate cancer, hepatocarcinoma, and renal cell carcinoma [103–107], suggesting its important role in cancer growth and progression, anoikis resistance, altered redox regulation, angiogenesis, and metastasis [80, 104, 108]. One potential link between ANGPTL4 and tumorigenesis is provided by hypoxia conditions, which represent a prominent feature of tumor microenvironment. Indeed, hypoxia induces overexpression of cyclooxygenase-2 (COX-2) by hypoxia-inducible factor-1 (HIF-1), an oxygen-sensitive transcriptional regulator [109–111], leading to the synthesis of prostanoids, especially prostaglandins PGE2. COX-2 is upregulated in recruited macrophages to trigger activation of other immune cells involved in antitumor response. Increased levels of PGE2 stimulate an intracellular signaling cascade leading to the induction of the ANGPTL4 expression and cANGPTL4 secretion [112]. Although most of studies have not explained the specific role played by ANGPTL4 as entire molecule or generated fragments, currently, several evidences suggested a prevalent activity of cANGPTL4. This fragment seems to be involved in “anoikis resistance,” which is a peculiar feature of metastatic cells acquiring ability to escape programmed cell death. cANGPTL4 interacts with beta-integrins to maintain an elevated ROS rate, inducing a redox-based survival mechanism that involves the activation of the SRC kinase and mitogen-activated protein kinase (MAPK) signaling pathways favoring cancer cell growth and survival [113].

As regards tumor angiogenesis, there are discordant data about the proangiogenic or antiangiogenic role of ANGPTL4. Several experiments showed that the ANGPTL4 increase by HIF-*α* stimulates the secretion of multiple proangiogenic factors regardless of vascular endothelial growth factor (VEGF). In this regard, one of the first evidences regards Kaposi's sarcoma, which is characterized by a deregulated angiogenesis process promoted by the release of proangiogenic molecules. Several in vitro and in vivo studies detected a significant ANGPTL4 upregulation in endothelial cells expressing a deregulated herpesvirus-8- (HHV-8- or KSHV-) encoded G protein-coupled receptor (vGPCR), which is considered a key factor in Kaposi's sarcoma tumorigenesis. ANGPTL4 inhibition has been associated with a significant decrease of neovascularization and vascular leakage in vitro and vGPCR-mediated tumorigenesis in vivo [114–116]. However, other studies have reported that ANGPTL4 exhibits an antiangiogenic role, inhibiting the proliferation, chemotaxis, and tubule formation of endothelial cells. One of such studies explored the effects of ANGPTL4 on the mouse epidermis through an in vivo neovascularization assay, revealing that ANGPTL4 decreased only VEGF-induced neovascularization, whereas it was not able to influence VEGF-independent neovascularization [27].

Finally, all these studies suggested that both proangiogenic and antiangiogenic effects of ANGPTL4 are reliable and strongly dependent on the related tumor microenvironment. Several evidences suggested that tumor microenvironment plays a crucial role in multiple steps of tumor development and progression, including drug resistance, immune-escaping, distant metastasis, and angiogenesis [117]. In particular, stromal cells are able to secrete multiple factors, including ANGPTL4, to enhance vasculature permeability in both lung and brain cancers [118].

Furthermore, as mentioned before, both the secretion and proangiogenic activities of ANGPTL4 are highly dependent on the vGPCR expression by the Kaposi Sarcoma tumor microenvironment. A recent study carried out on patients with uveal melanoma (UM) showed that ANGPTL4 secretion is regulated by HIF-1 and cooperates with VEGF in the angiogenesis promotion, supporting the potential benefit of a combined VEGF-ANGPTL4 inhibition to increase the efficacy of antiangiogenic treatments [119–121]. In addition, a recent work by Xin et al. [122] showed that HIF-1-induced upregulation of ANGPTL4 may promote vessel permeability in ischemic retinopathies, such as diabetic eye. All these evidences emphasize the need for further investigations about the posttranslational modifications that ANGPTL4 can undergo, to better understand how generated fragments could modulate pro- or anti-angiogenic events.

Some studies suggested that metastatic process seems to be pushed by the activation of human ANGPTL4 via TGF-*β*. This protein is an essential multifunctional cytokine involved in embryo development and tissue homeostasis but is secreted also in response to hypoxia and/or inflammation. In particular, it has been shown to induce an increase in human ANGPTL4 levels in breast cancer cells, by activating SMAD transcription factors, ultimately favoring the transendothelial migration of tumor cells through disruption of endothelial cell junctions [104, 123]. In hepatocellular carcinoma cells, ANGPTL4 also favors transendothelial migration and metastasis, through upregulation of vascular cell adhesion molecule-1 (VCAM-1) on endothelial cells, and stimulates the VCAM-1/integrin *β*1 signaling pathway, facilitating the cancer cell transendothelial extravasation to develop distant metastasis [124]. Another study concerning the role of ANGPTL4 in colorectal cancer patients positively correlated ANGPTL4 expression and venous invasion, which is considered the first step of metastatic process. However, the biological mechanism remains elusive [104]. Finally, a study only showed that ANGPTL4 may prevent tumor invasiveness and metastasis through modulation of both endothelial and tumor cell cytoskeleton organization [116]. Taken together, all these data suggest a potential prometastatic role for ANGPTL4, which of course needs to be deeply investigated in further studies, in order to elucidate the biological mechanisms underlying these processes. Since several studies suggested the involvement of ANGPTL4 in vascular permeability, angiogenesis, and inflammatory processes, ANGPTL4-modulating agents, such as PPARs, fatty acids, and specific drugs, could be useful for treatment of associated diseases [125].

## 5. ANGPTL4 as Potential Modulator of the Cross Talk between Metabolism and Cancer

Last decade has progressively evidenced two diseases, such as diabetes mellitus and obesity, as contributing factors to cancer onset and development. The first can favor tumor growth by increasing the availability of nutrients (e.g., glucose and FFA) or through alteration of the normal insulin signaling machinery, that causes an increase in blood lipid concentrations [126, 127]. Moreover, FFAs themselves can favor the instauration of oxidative stress through the formation of stress molecules such as reactive oxygen species (ROS), contributing to inflammation and tumor growth [128, 129]. The obesity could also be linked to cancer development through regulative mechanisms linked to adipokines and inflammatory cytokines [130, 131]. Indeed, obesity is characterized by accumulation of visceral adipose tissue that produces high quantities of inflammatory cytokines, mainly leptin, but also IL-1*β*, TNF-*α*, IL-8, and IL-6 [74, 132]. In adipocytes, PPAR*γ* activation has been associated with the upregulation of IRS-2 and CAP components of insulin pathway and hence to increased insulin sensitivity [133]. In this complex scenario, we speculated on the potential role played by some “pleiotropic” molecules, such as PPARs isoforms and ANGPTL4, in connecting lipid and glucose metabolism with cancer.

As previously reported, PPARs activation depends on the binding of different ligands. Among the PPAR ligands, there are natural and synthetic compounds, such as fibrates and thiazolidinediones, aimed at contrasting pathological conditions, including the dyslipidemic state (hypertriglyceridemia) and diabetes mellitus [134, 135].

An interesting work by Sethi et al. [136] showed that LDLs oxidation in endothelial cells causes their activation by PPAR agonists. Other PPAR agonists, such as bezafibrate, have been shown to directly improve insulin sensitivity through the activation of PPAR*γ* isoform [137].

As previously reported, the different PPAR isoforms are involved in lipid metabolism with different mechanisms of action, depending also on the tissue context in which they are [4, 83, 138–142]. Their deregulation can be evidenced in various tissue contexts. For example, an increase in PPAR*β*/*δ* expression was associated with a decreased lipid accumulation during a fat-rich diet in cardiac cells, whereas its overexpression in intestine was linked to colon cancer development [142]. Indeed, Sertznig et al. [4] showed that colon cancer cell activation depends on the stimulus induced by arachidonic acid, which leads to COX-2 upregulation and overproduction of prostaglandin PGE2. As previously reported, increased levels of PGE2 stimulate an intracellular signaling cascade, leading to the induction of ANGPTL4 expression and cANGPTL4 secretion [112]. Furthermore, macrophage PPAR*δ* induced by Th2 cytokines released by adipocytes has been shown to modulate the polarization of adipose tissue-resident macrophages, causing activation of an anti-inflammatory phenotype and consequently improving insulin sensitivity [143].

Also PPAR*γ* is involved in lipid metabolism. Similar to PPAR*β*/*δ* isoform, it regulates the activity of proteins like LPL [139, 140]. Due to this evidence, probably ANGPTL4, as well as other adipokines, is also a target of PPAR*γ*, acting as a mediator of lipid metabolism. Moreover, PPAR*γ* deregulation was detected not only in peripheral tissues linked to lipid metabolism, but also in inflammation and cancer [144]. Several evidences suggested that PPAR*γ* ligands may be potent inhibitors of angiogenesis mechanisms useful for anticancer therapy [145, 146]. Trombetta et al. [147] showed that fatty acids, such as docosahexaenoic acid (DHA), activate PPAR*γ* in cancer cells, leading to the inhibition of tumor development. Moreover, studies carried out on WAT, using long-chain monounsaturated fatty acids (LC-MUFAs), revealed a PPAR*γ* overexpression and a decrease of inflammatory markers in diabetes syndrome [148].

The increasing recognition of the dynamic entity of ANGPTL4 and its multifunctional role in different metabolic and nonmetabolic pathways, the expression network linking PPARs isoform to this angiopoietin-like protein, together with the recent evidences of involvement of PPAR in cancer, led to do some significant speculations on the potential molecular cross talk between these molecules, lipid metabolism, and cancer [149].

As previously reported, the ANGPTL4 expression depends on different stimuli, such as hypoxia and fasting and, finally, PPAR*β*/*δ*isoform induction [80]. Interestingly, recent evidences showed that ANGPTL4 expression is activated by PPAR*β*/*δ*, not only in adipose tissue but also in response to inflammation during wound healing [70]. Indeed, ANGPTL4 was defined as novel matricellular protein interacting with specific ECM proteins and integrins to facilitate cell migration during this event [54, 69]. This specific function could be linked to the C-terminal domain. Indeed, cANGPTL4 can activate *β*1 and *β*5 integrins, through their binding, in order to regulate cell migration via the focal adhesion kinase (FAK)/p21-activated kinase- (PAK-) signaling cascade [69]. Huang et al. [150] demonstrated that cANGPTL4 may induce vascular disruption through a direct and sequential association with integrin *α*5*β*1, VE-cadherin, and claudin-5, favoring metastasis. Other evidences showed that this protein fragment can bind to specific matrix proteins and delay their proteolytic degradation through the intervention of metalloproteinases [70]. Also, the endothelial ANGPTL4 secretion induced by tumor-released semaphorin 4D (SEMA4D) has been shown to modulate vascular permeability [151]. The absence of ANGPTL4 in macrophages has been shown to promote atherosclerosis, inducing foam cell formation and vascular inflammation [152]. Conversely, Georgiadi et al. [153] showed that ANGPTL4 overexpression reduces uptake of oxidized low-density lipoprotein (oxLDL) by macrophages and inhibits foam cell formation in murine models, consequently by counteracting the atherosclerosis development. More recently, Goh et al. [70] reported that ANGPTL4 upregulation after inflammatory stimulus determines the regulation of transcription factors involved in epidermal differentiation, such as protein kinase C (PKC) and activator protein-1 (AP-1).

Considering the similarities between the wound healing and cancer microenvironment, it becomes clear how the matricellular role of ANGPTL4 and its up- or downregulation can be translated in the neoplastic cellular context. As evidence of its role in cancer development, several scientific works showed a mRNA deregulation pattern associated with the tumor progression. Indeed,* ANGPTL4 *mRNA has been found to be upregulated in the perinecrotic areas of different tumor types [154, 155].

**Figure 5:**
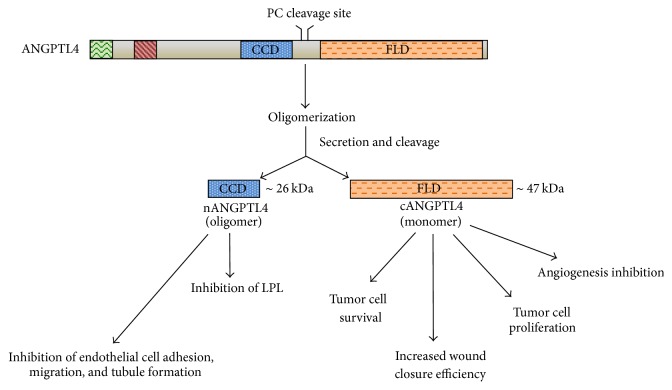
Different functional domains of ANGPTL4. Native full-length ANGPTL4 (flANGPTL4) is present as dimeric or tetrameric complexes. It can be processed to generate the N-terminal coiled-coil fragment (nANGPTL4) and COOH terminal fibrinogen-like domain (cANGPTL4), respectively. These protein fractions seem to have distinct roles depending on the tissue context.

A deeper investigation needs to be conducted on the roles played by different functional domains of ANGPTL4. As previously described, together with the full-length protein, nANGPTL4 and cANGPTL4 fragments can also be detected in plasma. Many scientific papers showed a different functional role for each of these protein domains. nANGPTL fragment seems to be mainly responsible for the regulation of lipid metabolism by inhibiting LPL activity, whereas a weaker effect in modulating triglyceride availability seems to be attributable also to flANGPTL4. Conversely, the cANGPTL4 fragment is involved in tumor cell growth, anoikis resistance, angiogenesis inhibition, and wound healing, depending on its interacting molecules [54] (Figure 5).

Interestingly, the different domains of ANGPTL4 and, consequently, their different roles are correlated to the tissue context in which they act. Obviously, this dependence is linked also to the different partners interacting with the various protein fragments. These evidences taken together led to speculate on the evolutionary benefit of having one protein with many functions correlated to distinct structural domains.

## 6. Conclusions

**Figure 6:**
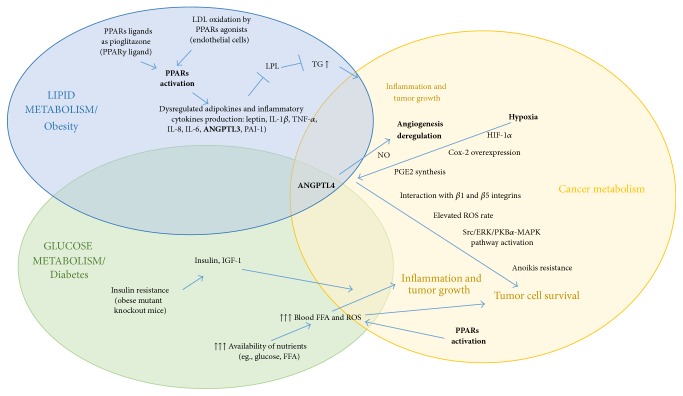
Potential cross talk between lipid/glucidic metabolism and cancer. Molecular pathways involved in communication between lipid/glucidic metabolism and cancer highlighting the key roles of PPAR and ANGPTL4. Abbreviations: ANGPTL3, angiopoietin-like 3; ANGPTL4, angiopoietin-like 4; Cox-2, cyclooxygenase-2; ERK, extracellular signal-regulated kinases; FFA, free fatty acids; HIF-1*α*, hypoxia-inducible factor-1 alpha; IGF-1, insulin-like growth factor-1; IL-1*β*, interleukin-1 beta; IL-6, interleukin-6; IL-8, interleukin-8; LDL, low-density lipoprotein; LPL, lipoprotein lipase; MAPK, mitogen-activated protein kinase; PAI-1, plasminogen activator inhibitor-1; PGE2, prostaglandin E2; PKB*α*, protein kinase B alpha; PPARs, peroxisome proliferator-activated receptors; ROS, reactive oxygen species; Src, V-SRC Avian Sarcoma (Schmidt-Ruppin A-2) Viral Oncogene; TG, triglycerides; TNF-*α*, tumor necrosis factor-alpha.

Abundant evidences opened the way to speculate a potential synergic role of PPARs and ANGPTL4 as key players in the cross talk between metabolic syndromes and cancer (Figure 6).

Indeed, as shown, (1) diabetes and obesity are important cancer comorbidity factors; (2) PPARs are involved in lipid and glucose metabolism; (3) PPAR*γ* and PPAR*β*/*δ* regulate the expression level of inflammatory cytokines and adipokines, such as leptin and ANGPTL4; (4) the deregulation of PPAR isoforms was detected in different types of cancer; (5) the ANGPTL4 expression depends on the PPAR stimulus. Considering all these points previously discussed, it would be legitimate to hypothesize that ANGPTL4 transcription regulation by PPARs constitutes a gateway between obesity, insulin sensitivity, and cancer.
